# Transforming the untransformable with knockout minicircles

**DOI:** 10.1002/mbo3.1374

**Published:** 2023-08-21

**Authors:** Oleksandra Rudenko, Laura Baseggio, Fynn McGuigan, Andrew C. Barnes

**Affiliations:** ^1^ School of Biological Sciences and Centre for Marine Science The University of Queensland Brisbane Queensland Australia; ^2^ School of Chemistry and Molecular Biosciences The University of Queensland Brisbane Queensland Australia

**Keywords:** *aip56* knockout, high‐efficiency transformation, minicircles, *Photobacterium damselae* subsp. *piscicida*, vector‐free allelic exchange

## Abstract

Gene inactivation studies are critical in pathogenic bacteria, where insights into species biology can guide the development of vaccines and treatments. Allelic exchange via homologous recombination is a generic method of targeted gene editing in bacteria. However, generally applicable protocols are lacking, and suboptimal approaches are often used for nonstandard but epidemiologically important species. *Photobacterium damselae* subsp. *piscicida* (*Pdp*) is a primary pathogen of fish in aquaculture and has been considered hard to transform since the mid‐1990s. Consequently, conjugative transfer of RK2/RP4 suicide vectors from *Escherichia coli* S17‐1/SM10 donor strains, a system prone to off‐target mutagenesis, was used to deliver the allelic exchange DNA in previous studies. Here we have achieved efficient electrotransformation in *Pdp* using a salt‐free highly concentrated sucrose solution, which performs as a hypertonic wash buffer, cryoprotectant, and electroporation buffer. High‐efficiency transformation has enabled vector‐free mutagenesis for which we have employed circular minimalistic constructs (knockout minicircles) containing only allelic exchange essentials that were generated by Gibson assembly. Preparation of competent cells using sucrose and electroporation/integration of minicircles had virtually no detectable off‐target promutagenic effect. In contrast, a downstream *sacB* selection apparently induced several large deletions via mobilization of transposable elements. Electroporation of minicircles into sucrose‐treated cells is a versatile broadly applicable approach that may facilitate allelic exchange in a wide range of microbial species. The method permitted inactivation of a primary virulence factor unique to *Pdp*, apoptogenic toxin AIP56, demonstrating the efficacy of minicircles for difficult KO targets located on the high copy number of small plasmids.

## Graphical abstract

We describe efficient vector‐free allelic exchange mutagenesis in *Photobacterium damselae* subsp. *piscicida*, potentially adaptable to any other bacterial species. It involves electroporation of minimalistic constructs containing only homology arms and selection markers, knockout minicircles, into cells rendered competent using concentrated sucrose solution. This approach is appropriate for genetic targets located on small plasmids and does not induce off‐target mutations, as demonstrated by knockout inactivation of *aip56* gene and sequencing of multiple mutagenesis clones, respectively.

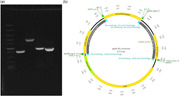

## 1 INTRODUCTION

*Photobacterium damselae* subsp. *piscicida* (*Pdp*) is a fish host‐adapted facultatively intracellular pathogen of the Vibrionaceae and a challenge to aquaculture throughout temperate marine waters (Andreoni & Magnani, 2014; Romalde, 2002). To date, there is no reliable control measure for photobacteriosis as both antibiotics and vaccines are ineffective or only partially effective against *Pdp* (Andreoni & Magnani, 2014; Håstein et al., 2005; Munang'andu, 2018). The pathogen possesses multiple virulence factors including a secreted toxin AIP56 that cleaves nuclear factor‐κB transcription factor, stopping the upregulation of the inflammatory genes and the downregulation of antiapoptotic genes in macrophages and neutrophils (Nunez‐Diaz et al., 2018; Pereira et al., 2014; Valderrama et al., 2019; do Vale et al., 2005). This critical virulence factor lacks homologs among known proteins, and the *Pdp* genome encodes a plethora of other not‐yet‐characterized in silico predicted proteins whose function is completely unknown (Baseggio et al., 2021). Gene inactivation is a robust way to determine or confirm a protein function (Nakashima & Miyazaki, 2014; Xu & Zhang, 2016), which, in pathogenic bacteria, can promote development of disease control measures such as protein subunit vaccines or nutritional therapy (Lu et al., 2021). Consequently, established or putative virulence factors are common gene knockout (KO) targets. The accuracy of the protein characterization hinges on the isogenicity of the KO mutant, because the phenotype may be affected by undesired (secondary, off‐target) mutations elsewhere in the genome (Johnson et al., 2003). Yet, reliable mutagenesis protocols are scarce for nonmodel organisms, and approaches demonstrated to have high rate of off‐target mutations are widely used (Babic et al., 2008; Ferrières et al., 2010; Strand et al., 2014).

Targeted gene KO in a wide range of bacteria can be achieved via allelic exchange by homologous recombination (Nakashima & Miyazaki, 2014; Xu & Zhang, 2016). In contrast to Red/ET recombination and CRISPR‐Cas9 methods that require introduction of plasmids for expression of gene‐editing enzymes (Nakashima & Miyazaki, 2014; Xu & Zhang, 2016), this approach relies on endogenous enzymatic machinery conserved across bacterial taxa (Michel & Leach, 2012). To generate the allelic exchange construct (AEC), the desired mutation (deletion, insertion, point mutation) is spliced between 0.5 and 1.5 kb regions homologous to sequences flanking the targeted locus (Nakashima & Miyazaki, 2014; Xu & Zhang, 2016). Next, the AEC is conventionally cloned into a plasmid vector containing marker genes for mutant selection, which is nonreplicating (suicide vector) or conditionally replicating in the recipient bacterial strain. Gibson assembly (GA) appears to be the most rapid and efficient approach to date, allowing simultaneous generation of the AEC and cloning into a plasmid vector in a single reaction (Huang & Wilks, 2017; Rudenko & Barnes, 2018). The next step is delivery of the allelic exchange plasmid, which ideally should be conducted via transformation of chemically competent or electrocompetent cells (Drury, 1994; Wirth et al., 1989). However, universal techniques to render cells competent are lacking and require optimization for many bacterial species and strains (Monk, 2012; Wang et al., 2020; Yildirim et al., 2016). Among epidemiologically important taxa, *Pdp* has been evidently poorly transformable since multiple conventional protocols were tested and shown inefficient (Cutrín et al., 1995, 2000). When a high‐efficiency transformation method is not available, an allelic exchange vector may be delivered by conjugation (Ferrières et al., 2010; Strand et al., 2014). To date, conjugative transfer of pir/RP4 suicide vectors from SM10/S17‐1 λ pir donor strains of *Escherichia coli* was the only method used to deliver the allelic exchange DNA in *Pdp* KO mutagenesis (Abushattal et al., 2022; Naka et al. 2005, 2007; Osorio et al., 2015). These donors contain the chromosomally integrated RP4::Mu conjugative transfer cluster (Simon et al., 1983), which was repeatedly shown to introduce off‐target mutations via Mu phage integration into the allelic‐exchange vector and/or the recipient's DNA, as well as Mu phage‐mediated and RP4‐mediated transfer and illegitimate integration of *E. coli* genes into the target host (Ferrières et al., 2010; Strand et al., 2014). Although alternative vectors and donors were designed (Babic et al., 2008; Ferrières et al., 2010; Strand et al., 2014), conjugation as a biological process has a mutagenic effect as the entrance of single‐strand DNA (ssDNA) via conjugation activates the stress response SOS that involves error‐prone replication and increased translocation/recombination of mobile elements (Baharoglu et al., 2010; Virolle et al., 2020). Further, whether DNA is delivered via conjugation or transformation, suicide vectors often integrate via illegitimate recombination (Johnson et al., 2003). In some cases, frequency of the off‐target mutations generated by suicide vectors can be sufficient for the latter to be used in random mutagenesis (Desomer et al., 1991).

In the present study, we have achieved high‐efficiency transformation in *Pdp* using a very simple protocol that utilizes only nonionic osmotic pressure (sucrose) to render the cells competent. Originally developed for streptococci (Framson et al., 1997), this salt‐free approach also performs well in Vibrio (Wang & Griffiths, 2009), and may potentially be a method of choice for a wide range of bacteria. High‐efficiency transformation has allowed us to mutagenize *Pdp* with nonreplicating DNA without conjugation, or even propagation of mutagenic DNA in *E. coli*. To further decrease the probability of secondary mutations, instead of conventional cloning of an AEC into a suicide vector, we have employed a vector‐free “minicircle” approach where homology arms were spliced with marker genes and circularized by GA. Minicircles were constructed and successfully integrated (single‐crossover mutants obtained) for two KO targets, a ubiquitous ssrA/smpB ribosome rescue system (Karzai et al., 2000) and AIP56 toxin, which is unique to Pdp (do Vale et al., 2005). In the latter case, double‐crossover mutants were also obtained with ease despite the expectation that *aip56* may be very hard to knock out due to its location on high copy number of small plasmids (Freitas et al., 2022). Our donor‐ and vector‐free allelic exchange based on transformation of sucrose‐treated competent cells with KO minicircles is a major improvement over previously used mutagenesis techniques in *Pdp*. The approach offers lower probability of secondary mutations and is potentially applicable to a broad range of bacterial species, including allegedly hard‐to‐transform species.

## 2 MATERIALS AND METHODS

### 2.1 Bacterial strains and routine culture

Strains were isolated from yellowtail kingfish (*Seriola lalandi*) during 2015–2016 disease outbreaks in South Australia and Western Australia, identified and deposited at the Department of Primary Industries and Regional Development (DPIRD), Western Australia as *Pdp* AS‐16‐0540‐1 and *Pdp* AS‐16‐0555‐7, and subsequently deposited as QMA0505 and QMA0506 at The University of Queensland (UQ), where isolates were sequenced (Baseggio et al., 2021). Bacteria were cultured at 25°C on Tryptic soy agar (TSA) or in Tryptic soy broth (TSB), unless otherwise specified.

### 2.2 Preparation of electrocompetent cells

Initially, we tested best‐performing conventional protocol in *Pdp* according to benchmarking performed by Cutrin et al. (1995) as described, except using TSB to grow cells instead of Brain heart infusion broth. Subsequently, cells were rendered competent using a salt‐free protocol developed for preparation of electrocompetent streptococci (Framson et al., 1997) with minor modifications. The overnight TSB cultures were diluted to 10^3^ colony‐forming unit (CFU)/mL (OD_600_ = 0.025 in Eppendorf BioPhotometer 6131) in 150–300 mL TSB, and grown to early exponential phase (10^4^–10^5^ CFU/mL; OD_600_ = 0.25–0.4). Harvested cultures were transferred to 50 mL tubes and chilled on ice; pelleted for 5 min at 2000*g*, 4°C; resuspended in 50–100 mL of ice‐cold 0.625 M sucrose solution in water (pellets combined) and pelleted for 10 min at 18 500*g*, 4°C; resuspended in 10 mL of ice‐cold 0.625 M sucrose (pellets combined), transferred into 15 mL tube, pelleted for 15 min at 18 500*g*, 4°C, and resuspended in 0.5–1 mL of the same buffer (or remaining supernatant if pellet was loose). This preparation was also attempted at room temperature using TSB supplemented with 1% salt to grow the culture. Competent cells were aliquoted by 50–90 μL into chilled 1.5 mL tubes and stored at – 80°C.

### 2.3 Transformation efficiency estimation

Competent cells were defrosted on ice, mixed with 100 ng of a plasmid carrying antibiotic resistance marker, pET‐28a(+), pLZ12spec, or pUC19, in 10 μL H_2_O, transferred to chilled 0.1 cm gap electro cuvettes (Sigma), and electroporated at the default P1 in Eppendorf Eporator (1250 V, 5 ms, 600 Ω). Cells were recovered in 0.5–1 mL of 0.25 M sucrose TSB for 2.5 h, 50 μL aliquots were spread on 75 μg/mL kanamycin (Km), 200 μg/mL spectinomycin, or 1 μg/mL ampicillin plates, and transformants were counted after 48 h. Viable cell counts were performed by Miles and Misra method (Miles et al., 1938) immediately after preparation, after defrosting, after electroporation, and after the recovery period.

### 2.4 Allelic exchange KO mutagenesis

#### 2.4.1 PCR used in mutagenesis

Primers used in allelic exchange mutagenesis of *aip56* and *ssrA‐smpB* are listed in Tables 1 and 2, respectively. They were designed using *Geneious Prime 2020.2.2* and Primer‐BLAST (NCBI). Annealing temperatures were determined by NEB Tm Calculator v1.13.0 (for this GA overhang parts were excluded from chimeric primers used to amplify the homology arms). Colony PCR screenings were performed using OneTaq® Quick‐Load® 2X Master Mix with Standard Buffer (NEB). Amplifications of GA fragments were performed using Q5 Hot Start High‐Fidelity 2X Master Mix (NEB). Products were visualized on 1% TAE gel stained with SYBR™ Safe DNA Gel Stain (Invitrogen) and size was determined using Fast DNA Ladder (NEB) marker.

**Table 1:** Primers used in *aip56* mutagenesis.

Purpose	Primer name	Sequence	Product (bp)
To amplify *kanR2* gene (selectable marker; kanamycin resistance)	Km_F	ATGAGCCATATTCAACGGGAA	816
	Km_R	TTAGAAAAACTCATCGAGCATC	
To amplify *sacB* transcript (counter‐selectable marker; sucrose sensitivity)	sacB_F	ACAAAGTCATCGGGCATTATC	2085
	sacB_R	CGGCTGACATGGGAATTCTG	
To amplify *aip56* upstream homology arm and splice it with *sacB* (F primer overhang) and *kanR2* (R primer overhang)	aip56_up_F	gttaaaaaggatcagaattcccatgtcagccg	887
		TTTGTTGTCCGCTGTTACTTG	
	aip56_up_R	cctagagcaagacgtttcccgttgaatatggctcat	
		GATTATTGAGTATTTTTTCACTGTGA	
To amplify *aip56* downstream homology arm and splice it with *kanR2* (F primer overhang) and *sacB* (R primer overhang)	aip56_down_F	attgcagtttcatttgatgctcgatgagtttttctaa	924
		TTCGATGTAATTCTGCTCTG	
	aip56_down_R	tttatgttcagataatgcccgatgactttgt	
		GCATCGTTAAGGTCATGTGT	
To verify crossover in upstream homology region	out_ aip56_F	ACGTAATGTGTCGCCCAACT	2755
	Km_R	TTAGAAAAACTCATCGAGCATC	
To verify crossover in downstream homology region	Km_F	ATGAGCCATATTCAACGGGAA	2673
	out_aip56_R	AGCGTTTTCTATACTGTTTTTGGTA	
To screen for *aip56* deletion	aip56_F	TCACGTTACAGGCTCTAGTG	388
	aip56_R	GCATTCAACTGAACTGTCGG	
To amplify across the *aip56* allelic exchange region	out_aip56_F	ACGTAATGTGTCGCCCAACT	4656 in KO
	out_aip56_R	AGCGTTTTCTATACTGTTTTTGGTA	5335 in WT

*Note*: Lowercase letters indicate Gibson assembly overhangs.

Abbreviations: KO, knockout; WT, wild type.

**Table 2:** Primers used in *ssrA‐smpB* mutagenesis.

Purpose	Primer name	Sequence	Product (bp)
To amplify *kanR2* gene (selectable marker; kanamycin resistance)	Km_F	ATGAGCCATATTCAACGGGAA	816
	Km_R	TTAGAAAAACTCATCGAGCATC	
To amplify *sacB* transcript (counter‐selectable marker; sucrose sensitivity)	sacB_F	ACAAAGTCATCGGGCATTATC	2085
	sacB_R	CGGCTGACATGGGAATTCTG	
To amplify *ssrA* upstream homology arm and splice it with *sacB* (F primer overhang) and *kanR2* (R primer overhang)	ssrA_up_F	gttaaaaaggatcagaattcccatgtcagccg	1137
		GGGCCACGAAAAACCTGAAC	
	ssrA_up_R	cctagagcaagacgtttcccgttgaatatggctcat	
		GTTGACCTCTTAAGTCTCTGTTGC	
To amplify *ssrA* downstream homology arm and splice it with *kanR2* (F primer overhang) and *sacB* (R primer overhang)	ssrA_down_F	attgcagtttcatttgatgctcgatgagtttttctaa	1407
		AATTCGTATACGAAGACGTCC	
	ssrA_down_R	tttatgttcagataatgcccgatgactttgt	
		TGAGTTTCGCAATGACAGCA	
To verify crossover in upstream homology region	out_ssrA_F	TGGAACCCCAATGCTAGTTGA	2019
	Km_R	TTAGAAAAACTCATCGAGCATC	
To verify crossover in downstream homology region	Km_F	ATGAGCCATATTCAACGGGAA	2657
	out_ssrA_R	CACGGTGGTGAGGTGACTC	
To verify ssrA‐smpB deletion	ssrA_F	CTGCTCAGAGCCTGCTATCC	145
	ssrA_R	CAGACACGCCATGCAAAGAC	
	smpB_F	TAACAGGCGCAGTAAACCGT	156
	smpB_R	CCTTATCACGCTGCCAGTCA	
To amplify across the allelic exchange region (not used)	out_ssrA_F	TGGAACCCCAATGCTAGTTGA	3860 in KO
	out_ssrA_R	CACGGTGGTGAGGTGACTC	4019 in WT

*Note*: Lowercase letters indicate Gibson assembly overhangs.

Abbreviations: KO, knockout; WT, wild type.

#### 2.4.2 Construction of the allelic exchange “KO minicircles”

**Figure 1:**
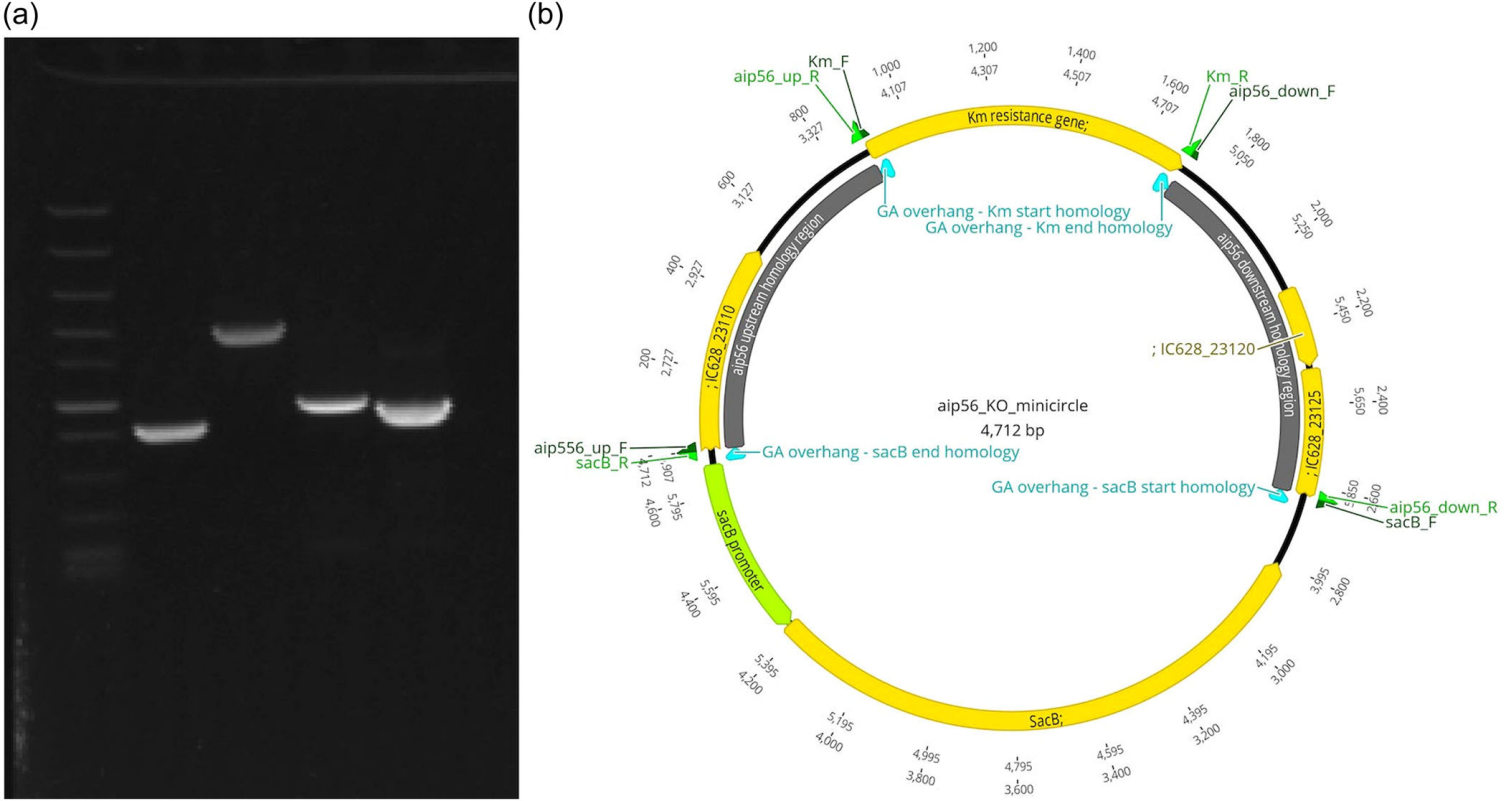
*aip56* knockout (KO) minicircle: (a) Amplicons of the assembly fragments: Lane 1—Fast DNA Ladder (NEB) marker with 10, 5, 3, 2, 1.5, 1 (reference), 0.766, 0.5, 0.3, 0.15, and 0.05 kb bands; Lane 2—*kanR2* gene sequence (Km_F/R); Lane 3—*sacB* transcript sequence (sacB_F/R); Lane 4—*aip56* upstream homology arm (aip56_up_F/R); Lane 5—*aip56* downstream homology arm (aip56_down_F/R). (b) Schematic representation of the assembled minicircle. F, forward primer binding site; GA overhang, Gibson assembly overhang; Km, kanamycin; KO, knockout; R, reverse primer binding site.

**Figure 2:**
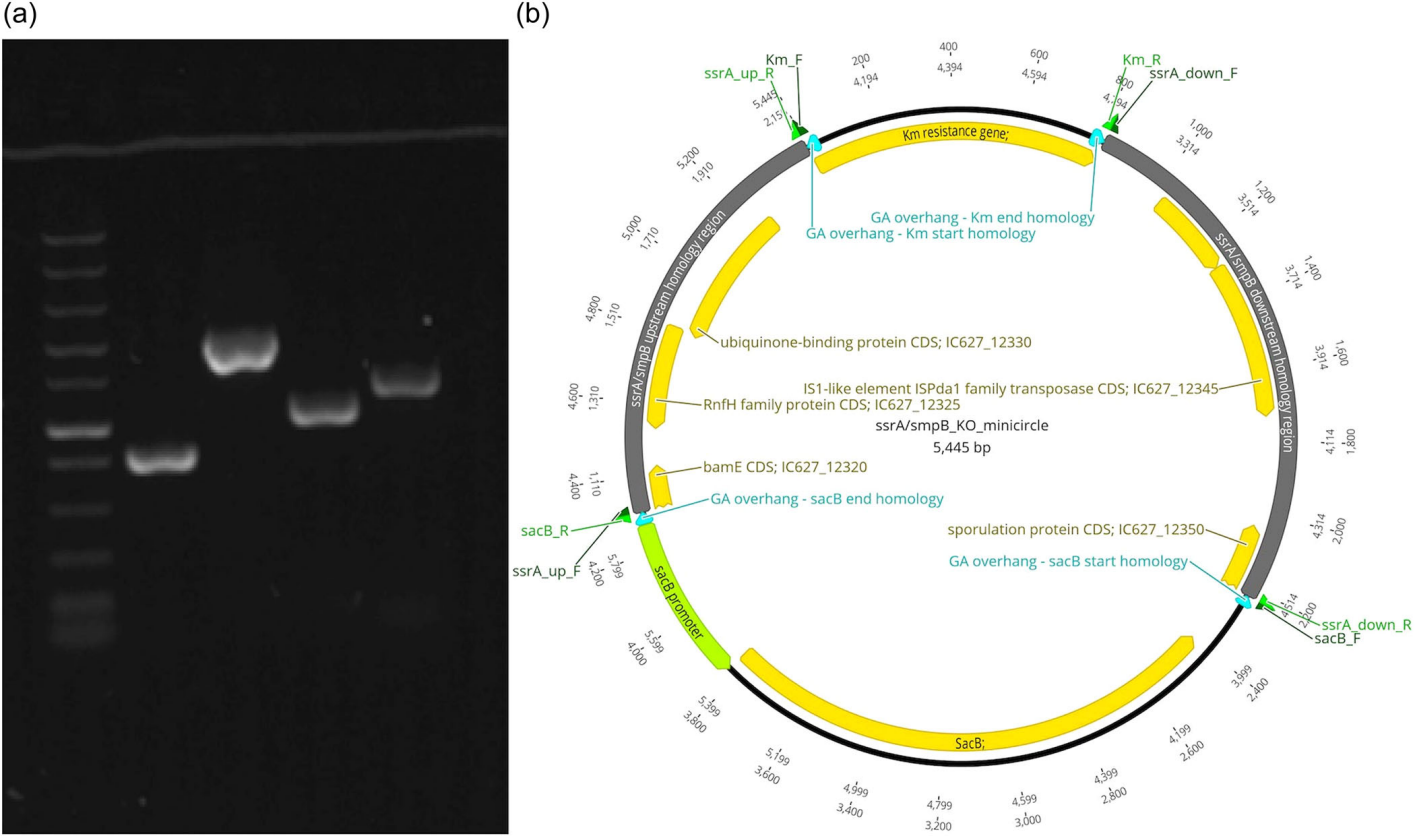
*ssrA/smpB* knockout (KO) minicircle: (a) Amplicons of the assembly fragments: Lane 1—Fast DNA Ladder (NEB), marker with 10, 5, 3, 2, 1.5, 1 (reference), 0.766, 0.5, 0.3, 0.15, and 0.05 kb bands; Lane 2—*kanR2* gene sequence (Km_F/R); Lane 3—*sacB* transcript sequence (sacB_F/R); Lane 4—*ssrA/smpB* upstream homology arm (ssrA_up_F/R); Lane 5—*ssrA/smpB* downstream homology arm; (ssrA_down_F/R). (b) Schematic representation of the assembled minicircle. F, forward primer binding site; GA overhang, Gibson assembly overhang; Km, kanamycin; KO, knockout; R, reverse primer binding site.

Circular minimalistic AECs (“KO” minicircles) for inactivation of *aip56* and *ssrA*/*smpB* genes were designed to contain homology arms spliced between selectable and counterselectable marker genes (Figures 1b and 2b). *kanR2* gene (selectable marker conferring Km resistance; expressed under target gene transcription elements) and *sacB* gene with promoter and terminator (counterselection marker conferring sucrose sensitivity) were amplified using Km_F/R and sacB_F/R primer sets (Tables 1 and 2), from 0.5 ng of pET‐28a(+) (Addgene; #2526) and pRE107 (Addgene; #43829) plasmids, respectively. The pET‐28a reaction was treated postamplification with *Dpn*I (NEB) to eliminate transformation background produced by pET‐28a(+) plasmid used as a PCR template. The latter was extracted immediately before amplification to ensure that methylation required for *Dpn*I restriction was retained. Homology regions (0.9–1.4 kb sequences upstream and downstream from *aip56* and *ssrA‐smpB* targets) were amplified from *Pdp* strain QMA0505 gDNA (CP061854‐60) using chimeric primers containing GA overhangs homologous to the ends of *kanR2* and *sacB* (aip56_up_F/R, aip56_down_F/R, ssrA_up_F/R, and ssrA_down_F/R reactions; Tables 1 and 2). Fragments (Figures 1a and 2a) were spliced into KO minicircles (Figures 1b and 2b) using NEBuilder HiFi DNA Assembly Cloning Kit (NEB) in 1:1:1:1 molar ratio in 30 μL reactions for 2 h (total of 0.75 pmol DNA, maximum recommended input). Schematic representations of minicircles in Figures 1b and 2b were created in Geneious Prime 2020.2.2.

#### 2.4.3 Minicircle transformation and selection of *aip56* and *ssrA‐smpB* meroploids

GA reactions of *aip56*‐ and *ssra/smpB*‐KO minicircles were purified using AMPure XP magnetic beads (Beckman Coulter), eluted in 20 μL dH_2_O, and quantified by Qubit. Approximately 0.7 μg of purified minicircles in 10 μL was used to transform QMA0505 (UQ)/AS‐16‐0540‐1 (DPIRD) strain of *Pdp* (Baseggio et al., 2021) as described (subsection 2.3), except for the extension of the recovery period to 4 h to allow for chromosomal integration of *kanR2* for expression, and the exclusion of 0.25 M sucrose from the recovery medium, as it is lethal after integration of *sacB*‐containing minicircles. Transformation with ssDNA was also attempted. For this, half of the purified assembly reactions (10 μL) were denatured at 95°C for 2 min and snap cooled. Single‐crossover mutants (meroploids containing integrated minicircles) were selected on 75 μg/mL Km and confirmed by colony PCR screening for the presence of recombination (crossover) in upstream or/and downstream homology regions—amplification across the homology regions where one primer binds to *kanR2* sequence and the second primer binds to a sequence outside the homology (out_aip56_F/Km_R, Km_F/out_aip56_R, out_ssrA_F/Km_R, and Km_F/out_ssrA_R reactions; Tables 1 and 2).

#### 2.4.4 Selection of *aip56*‐ and *ssrA‐smpB* KO mutants

To select double‐crossover clones (KO strains) of *aip56*, single‐crossover mutants were grown to saturation in nonsupplemented TSB, subcultured once, and plated onto agar containing 75 μg/mL Km lethal to wild type (WT) and 15% w/v sucrose (lethal to meroploids). Emergent clones were picked, streaked on the fresh plates, subcultured one more time, and screened for the loss of target genes by colony PCR using an *aip56*‐specific primer set (aip56_F/R; Table 1) (Abushattal et al., 2020). *aip56* deletion was further confirmed by amplification across the allelic exchange region yielding a shorter product compared to the WT strain (out_aip56_F/R reaction; Table 1). Single‐crossover mutants of ssrA‐smpB were grown in multiple liquid culture conditions: TSB, TSB supplemented with 7.5% or 15% sucrose, TSB supplemented with  7.5% or 15% sucrose and 75 μg/mL Km, with decreased agitation or stationary. Most attempts included multiple cultures ranging from 10 to 96. These were plated on 75 μg/mL Km/15% sucrose plates or 50 μg/mL Km/7.5% sucrose plates, and emergent colonies were screened using *ssrA*‐ and *smpB‐*specific primer sets (ssrA_F/R, smpB_F/R; Table 2).

#### 2.4.5 Simultaneous selection of *aip56*‐KO clones and “revertant” clones from multiple independent cultures

Multiple *aip56* KO clones and clones that reverted to WT genotype (“revertants”) were selected in parallel from independent broth cultures of the single meroploid clone as follows: QMA0786 meroploid used to generate the initial *aip56* KO clone QMA0648 was recovered from the frozen stock on 75 μg/mL Km agar and individual colonies were picked to initiate four independent cultures in nonsupplemented TSB, subcultured once, and spread on 15% sucrose plates. Individual colonies were picked and patched onto both 15% sucrose and 75 μg/mL Km/15% sucrose plates. Suspected KO clones (growth on both plates) and clones that reverted to the WT (no growth on Km plates) were PCR‐screened with aip56_F/R and KmF/R primer sets (Table 1).

### 2.5 Sequencing of mutagenesis clones and detection secondary of mutations

To detect secondary mutations, DNA was extracted from selected clones generated during *aip56* mutagenesis (QMA0648, QMA0780‐89) using Quick‐DNADNA Miniprep Plus Kit (Zymo Research). Short‐read (Illumina) library preparation and sequencing were performed at the University of Queensland Sequencing Facility (UQ, Brisbane, Australia). Sequencing libraries were prepared using the Nextera XT DNA Library Prep Kit (Illumina), purified with 30 µL of AxyPrep Mag PCR Clean‐up beads (Axygen), quantified on the PerkinElmer LabChip GX with the DNA High Sensitivity Reagent Kit (PerkinElmer; CLS760672), and pooled in an equimolar ratio. Sequencing was performed using the Illumina NextSeq500 (NextSeq control software v4.0.0/Real‐Time Analysis v2.11.3). The library pool was diluted and denatured according to the standard NextSeq protocol and sequenced to generate paired‐end 151 bp reads using a 300‐cycle NextSeq500/550 Mid Output Reagent Kit v2.5 (Illumina). After sequencing, fastq files were generated using bcl2fastq2 (v2.20.0.422). For better comparability/assessment of the initial frequency of mutation, WT strain QMA0505 was also resequenced along with mutagenesis clones as Illumina reads for it's published genome C06185‐460 were obtained using different sequencing kit and instrument (Baseggio et al., 2021). Reads were trimmed using fastp 0.23.2 and paired during export into Geneious Prime 2023.0.4. Using the latter software, reads were mapped to the CP061854‐60 genome (Baseggio et al., 2021) using Geneious assembler under default settings. Variants were identified using “Find variations/SNPs” command with minimum coverage set to 30 and minimum variant frequency set to 0.9. Where annotations differed between the polymorphism tracks, alignment of the reads was examined for strains/contigs lacking the identified variants, and the latter were assigned if lower than set coverage was the reason for the variant not being annotated. Where lower variant frequency was the reason, reads with and without the variant were counted and polymorphism was assigned for over 0.6% frequency cases. QMA0505 read alignment at each variant position was examined to identify whether mutation is *de novo*. Regions of no read coverage indicating putative large deletions were identified using “Find High/Low coverage” command with a number of sequences under the low coverage flag set to “0.” Where large regions of no coverage were found long‐read (Nanopore) sequencing was carried out to confirm the putative large deletions. For the latter, libraries were prepared using Rapid Barcoding Kit 24 V14 (SQK‐RBK114.24) and sequenced on a MinION Mk1C device equipped with FLO‐MIN114 (R10) flow cell. Nanopore reads were assembled using Flye v2.9, annotated with Prokka v1.12, and exported to Geneious. Regions containing putative deletions were extracted from lon‐gread assemblies and the CP061854‐60 genome sequence, and aligned using Clustal Omega.

## 3 RESULTS AND DISCUSSION

### 3.1 Salt‐free preparation of electrocompetent *Pdp*

We aimed to inactivate two targets via gene KO mutagenesis: AIP56 toxin and SsrA‐SmpB ribosomal rescue system, an established and recently proposed virulence factors in *Pdp*, respectively (Baseggio et al., 2021). For the purpose of obtaining isogenic KO mutants, we wanted to avoid mating with SM10/S17‐1 λ pir donor strains of *E. coli* due to high off‐target mutation rate produced by RP4::Mu conjugative transfer cluster (Ferrières et al., 2010; Strand et al., 2014). Transformation is a more straightforward and rapid approach compared to conjugation since allelic exchange DNA is delivered directly to the target organism. Furthermore, ssDNA entering the recipient's cell via conjugation activates bacterial stress response SOS which induces error‐prone recombination (Baharoglu et al., 2010; Virolle et al., 2020). Consequently, transformation with double‐stranded DNA (dsDNA) is likely to offer lower probability of secondary mutations. For transformation via electroporation, cells are typically washed in hypertonic solutions, as high osmotic pressure stabilizes the cell membrane and decreases cell lysis during electroporation. Most protocols rely on salt ions to create high osmotic pressure; however, salts cause arcing during electroporation and must be washed off in advance. Washed cells are typically resuspended in an electroporation solution containing glycerol that prevents freezing damage and allows storage (Wirth et al., 1989). However, many bacterial species and strains are not readily transformable by standard methods (Aune & Aachmann, 2010), and high transformation efficiency is required for the delivery of nonreplicating DNA in allelic exchange mutagenesis. Case in point, previous attempts to render *Pdp* cells electrocompetent using the outlined basic approach were not particularly successful. Multiple conditions were evaluated by Cutrin et al. in 1995, and according to the authors, excessive cell lysis occurred when cells were grown in a salty medium (standard for marine species) and prepared using low ionic strength buffers, and arcing occurred when cells were prepared using “isotonic for the bacteria” (i.e., high ionic strength) buffers (Cutrín et al., 2000). Of all the evaluated conventional methods, the highest efficiency of 9.8 × 10^2^ CFU/μg DNA was achieved with a laborious multiple‐buffer multiple‐reagent protocol immediately after preparation without defrosting/prior storage (Cutrín, 1995). We attempted the same procedure in our laboratory and obtained around 10^2^ CFU/μg transformation efficiencies with pET‐28a(+) and pUC19 plasmids.

Since higher efficiencies are required for transformation with nonreplicating DNA, we attempted a rapid and straightforward single‐buffer protocol originally developed by Dunny et al. (1991) for gram‐positive bacteria. The method was subsequently applied by Framson et al. (1997) for group B streptococci and optimized to contain no salt in the buffer, which rendered this single‐buffer protocol to be also a single reagent. The sole ingredient in the wash/electroporation buffer is 0.625 M sucrose, which works as a hypertonic stress agent (nonionic osmotic pressure), electroporation medium, and cryoprotectant. Following the above protocol, we have repeatedly achieved transformation efficiencies of 10^6^–10^7^ CFU/μg DNA in *Pdp* using pET‐28a(+) and pUC19 vectors, and somewhat lower efficiencies of 10^5^–10^6^ CFU/μg DNA were obtained with streptococcal pLZ12spec vector. Notably, these efficiencies were estimated with defrosted cells previously stored at −80°C. We performed the protocol with only minor modifications: TSB was used instead of Todd–Hewitt broth (used to grow streptococci) with no supplementation with glycine, which is used to weaken cell walls in gram‐positive bacteria but unnecessary for gram‐negative preparations. Notably, supplementation of the growth medium with salt used to culture marine bacteria including *Pdp* was also routinely omitted for competent cell preparation to reduce the chances of arcing. Yet, there were no arcing events or reduction in transformation efficiency in several preparations from cultures grown in TSB supplemented with 1% salt (1.5% final salt concentration). This suggests that the method can potentially be used for strictly halophilic bacteria if salts from the growth medium are efficiently washed off by the sucrose solution. There was only minor reduction in the number of viable cells after defrosting and a 10‐fold reduction after the electroporation. We have also tried room‐temperature preparation and transformation, which may be used as a more convenient alternative to traditional ice‐cold technique (Tu et al., 2016). This approach was functional but 10–100 times less efficient.

We have efficiently transformed sucrose‐treated electrocompetent *Pdp* with minimalistic AEC generated by GA (KO minicircles) targeting *aip56* and *ssrA‐smpB* loci (see subsections 3.2 and 3.3), which demonstrates that achieved transformation efficiency is sufficient for allelic exchange mutagenesis with nonreplicating DNA. The most prominent difference between this protocol and the conditions evaluated by Cutrín et al. (1995) is the composition of the hypertonic (osmotic stress) buffer. The former has increased sucrose concentration and a complete absence of salt; thus, the osmotic pressure created is completely nonionic. This is highly advantageous because any unwashed salts can cause arcing, while washing/resuspension steps require extra time and handling, and may lower the stabilizing effect on the membranes created by a hypertonic buffer. Further, divalent cations from salts may be used as cofactors by nucleases targeting the transforming DNA (Wang & Griffiths, 2009). Indeed, salt‐free preparations dramatically improve transformation efficiency in *Vibrio parahaemolyticus*, which was explained by deactivation of extracellular DNase abundantly secreted by Vibrio (Wang & Griffiths, 2009). In contrast, highly concentrated sucrose solution may be conveniently used as both hypertonic buffer and electroporation buffer as it creates nonionic osmotic pressure, which efficiently stabilizes cell membranes without adversely affecting the electroporation or augmenting DNase activity. Moreover, it has cryoprotective properties, which allow freezing of competent cells without the addition of glycerol.

### 3.2 “KO minicircles”: circular AECs generated by GA

Nonreplicating plasmids (suicide vectors) are most often used to deliver mutagenic DNA constructs in allelic exchange mutagenesis. Yet, suicide vectors are notorious for illegitimate integration into host chromosomes and plasmids and other kinds of off‐target mutagenesis (Johnson et al., 2003). Illegitimate recombination is very common in bacteria, and it is facilitated in plasmid sequences by the abundance of repeats and common/universal motifs (Desomer et al., 1991; Li et al., 2018; Oliveira et al., 2010; de Vries & Wackernagel, 2002). Remarkably, some suicide plasmids illegitimately integrate at frequencies sufficient for random mutagenesis experiments (Desomer et al., 1991). In some circumstances, plasmid backbones may be necessary, such as when transformation is not available, so DNA is delivered by conjugation, or when transformation is low in efficiency, so very large quantities of DNA are required the plasmid is replicated in and extracted from *E. coli* for electroporation. However, whenever high‐efficiency transformation and other factors permit, vector‐free KO approach should be considered as an alternative offering lower off‐target mutagenesis probability. Vector‐free AECs in previous studies were Fusion PCR products circularized by ligation (Gomaa et al., 2017) and linear GA products (Wu et al., 2019). Here we combined these two approaches: we used GA as a more convenient alternative to Fusion PCR technique (Huang & Wilks, 2017; Rudenko & Barnes, 2018), but employed it to generate circular constructs/products, as they are more stable compared to linear DNA. The constructs, here dubbed “KO minicircles,” were generated to knock out  *aip56* and *ssrA‐smpB* ribosomal rescue loci (Figures 1 and 2). Minicircles were designed for marked nonpolar deletions and contained four fragments assembled in the following order: ‐> upstream homology arm ‐> selectable marker gene (*kanR2*, Km resistance) ‐> downstream homology arm ‐> counter‐selectable marker transcript (*sacB*, sucrose sensitivity) ‐>. In‐frame insertion of the selectable marker gene (without transcription elements) in the place of the KO target does not cause polar effects, facilitates mutant selection, and helps to maintain purity of the culture. Alternatively, if unmarked deletion is desired, a selectable marker gene (with transcription elements) can be placed outside the homology regions along with a counter‐selectable marker. Overall, the minicircle approach is exceptionally versatile and may be used with any selectable and counter‐selectable marker/s, and for other genetic modifications including site‐directed mutagenesis and gene knock‐in, e.g., insertion of the WT gene for the KO rescue.

### 3.3 *aip56* and *ssrA‐smpB* mutagenesis: Selection of single‐crossover mutants

**Figure 3:**
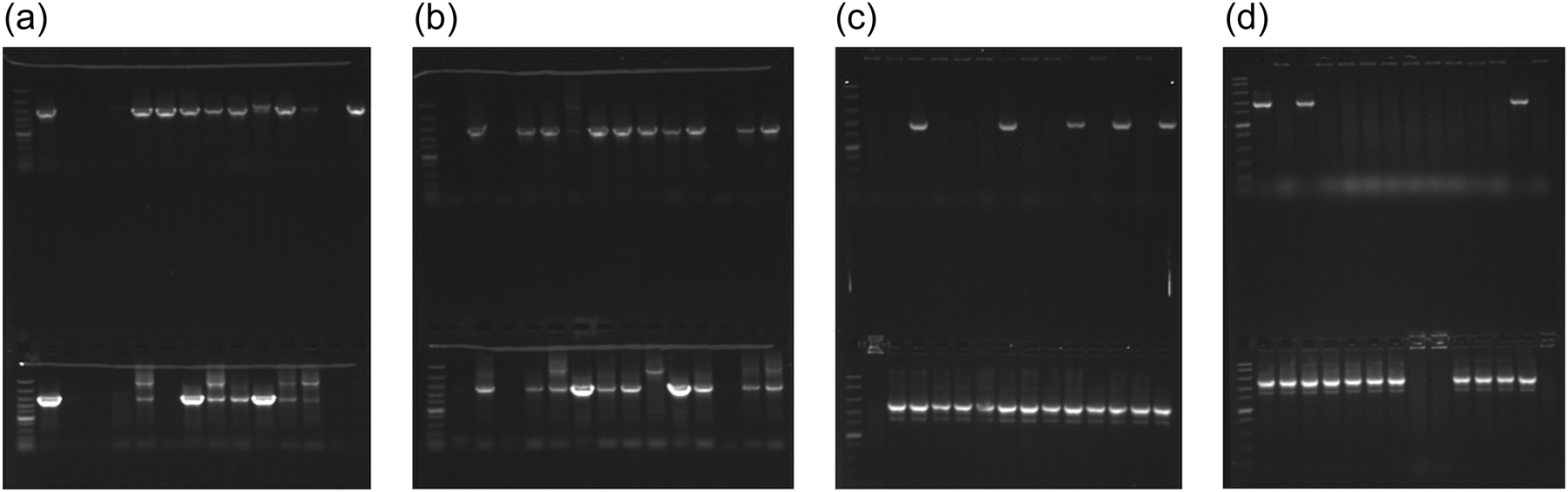
Colony PCR screening after transformation with double‐stranded (a) and denatured (b) assembly reactions of *aip56* minicircle and double‐stranded (c) and denatured (d) assembly reactions of *ssrA/smpB* minicircle—amplification from *kanR2* to genomic sequence outside the homology arms confirming integration of minicircles. The top parts of the gels show integration via crossover in the upstream homology region (a, b—out_aip56_F/Km_R; c, d—out_ssrA_F/Km_R), the bottom parts of the gels show integration via crossover in the downstream homology region (a, b—Km_F/out_aip56_R; c, d—Km_F/out_ssrA_R), amplicons in corresponding top and bottom parts of gels are derived from the same clone, marker in the first lane of the gels is Fast DNA Ladder (NEB) with 10, 5, 3, 2, 1.5, 1 (reference), 0.766, 0.5, 0.3, 0.15, and 0.05 kb bands.

High frequency of single‐cross mutants (meroploids with integrated minicircles) in four transformations (*aip56* and *ssrA‐smpB* minicircles, dsDNA, and ssDNA each) provided evidence that: (1) transformation efficiency is sufficient for delivery of nonreplicating allelic exchange vectors or vector‐free constructs; (2) KO minicircles generated by GA readily integrate into chromosomes and plasmids; and (3) electroporation with KO minicircles is a straightforward and efficient way to deliver allelic exchange DNA. Only ~0.7 μg of assembly products (0.65 μg of *aip56* and 0.78 μg of *ssrA‐smpB* construct DNA) were used in mutagenesis, which were delivered either double‐stranded or denatured. Many Km‐resistant clones were obtained in all transformations, although around two times more in transformations with ssDNA (Table 3). Randomly chosen selected colonies were PCR‐screened for the presence of crossover using primer sets where one primer binds to the Km sequence and the second one binds to the genomic sequence outside the homology region. Over 70% of PCR‐screened colonies yielded products of expected size (Figure 3 and Table 3), which unambiguously showed successful integration of minicircles. In the case of *aip56*, both homology arms recombined equally well (Figure 3a,b), while, in the case of *ssrA*, the upstream region was apparently less recombinable (Figure 3d,c).

**Table 3:** Selection of *aip56* and *ssrA* KO mutants after transformations with ds and denatured (ss) minicircles.

Number of clones	ds‐aip56	ss‐aip56	ds‐ssrA	ss‐ssrA
Selected on kanamycin plates	32	54	60	112
PCR‐screened for minicircle integration (via single crossover)	14	14	14	14
PCR‐positive for minicircle integration (confirmed single‐crossover mutants)	10	11	13	11
Independent nonselective broth cultures	8	Not used	8	Not used
Selected on kanamycin/sucrose plates	30–200	‐	None	‐
PCR‐screened for deletion of the target gene from each selection	6 per culture (×8)	‐	‐	‐
PCR‐negative for the target gene (confirmed KO mutants)	3/3/0/3/5/1/2/0 (out of six screened)	‐	‐	‐

Abbreviations: ds, double‐stranded; KO, knockout; ss, single‐stranded.

### 3.4 *aip56* and *ssrA‐smpB* mutagenesis: Selection of double‐crossover mutants

**Figure 4:**
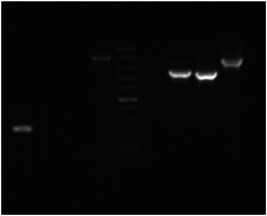
PCR confirmation of *aip56* knockout: DNA marker in Lane 5—Fast DNA Ladder (NEB) with 10, 5, 3, 2, 1.5, 1 (reference), 0.766, 0.5, 0.3, 0.15, and 0.05 kb bands, on the left from the marker are amplicons derived from wild‐type strain gDNA, on the right from the marker are amplicons of corresponding reactions performed using knockout strain gDNA. Lanes 1, 5—*aip56*‐specific reaction (aip56_F/R). Lanes 2, 6—Amplification across the upstream homology arm (out_aip56_F/Km_R). Lanes 3, 7—Amplification across the downstream homology arm (out_Km_F/out_aip56_R). Lanes 4, 8—Amplification across allelic exchange region (out_aip56_F/R).

To select double‐crossover mutants, meroploids obtained from dsDNA‐minicircle transformations were used, as more likely to be isogenic (Baharoglu et al., 2010; Virolle et al., 2020). Multiple *aip56* KO clones were obtained with ease after two subcultures of single‐crossover mutants in nonselective broth (TSB) and plating on agar supplemented with sucrose (lethal to single crossovers) and Km (lethal to WT). Randomly chosen clones were PCR‐screened for the loss of *aip56* gene (Table 3). Several *aip56‐*negative clones were further confirmed to be KO mutants by amplification across the allelic exchange region yielding a shorter product compared to WT strain (Figure 4). PCR screening for *aip56* deletion showed that many meroploids were escaping sucrose counterselection, which is often weak and inefficient (Cianfanelli et al., 2020; Lazarus et al., 2019), despite omission of salt supplementation in selective plates. Since salt inhibits *sacB* expression (Blomfield et al., 1991), the latter marker may be suboptimal for slight halophiles such as *Pdp* and completely inappropriate for obligate halophiles. Nonetheless, double‐crossover mutants were selected from 75% of the broth cultures and accounted for 16.65%–83% of the screened colonies (Table 3). Such a success rate was unexpected since *aip56* is encoded on a highly abundant, small (<10 kB) plasmid that was anticipated to be hard to eliminate (Freitas et al., 2022). Potentially, *aip56* KO would have been harder, perhaps impossible, to achieve using a suicide vector, since integration of a long vector backbone sequence with plasmid replication elements is likely to compromise the *aip56* plasmid stability and/or replication . AIP56 is a known toxin characterized at the protein level, which is a primary virulence factor of *Pdp* causing apoptosis in professional phagocytic cells (Pereira et al., 2014; do Vale et al., 2005). However, isogenic KO mutants lacking the gene have not been characterized yet, which hampers the research aiming at a complete understanding of *Pdp* pathogenicity (Freitas et al., 2022).

Although both *ssrA‐smpB* homology arms were efficiently recombining (Figure 3 and Table 3), double‐crossover mutants were not obtained when followed as per *aip56* KO selection despite multiple subsequent attempts involving up to 24 independent single‐crossover cultures. Also, based on the hypothesis that *ssrA* KO mutant (if viable) would be generally impeded in growth and more sensitive to oxidative stress, antibiotics, and high sugar concentrations, we tried the following modifications to the selective conditions: stationary broth cultures, broth cultures with sucrose and sucrose/Km, and lower Km and sucrose concentrations on selective plates, but still we were unable to select *ssrA‐*negative clones. Notably, supplementation of broth cultures with sucrose and lower sucrose concentration on plates dramatically increased the number of single‐crossover mutants escaping *sacB* counterselection, and thus, decreased chances to detect double‐crossover mutants, if any were present at minor frequency. Thus, either some alternative counter‐selectable markers are required to obtain *ssrA* KO mutant, or *ssrA* may be essential in *Pdp*. Indeed, *ssrA‐smpB* is essential in obligatory intracellular pathogens (Karzai et al., 2000), and *Pdp* causes primarily intracellular infections and shows relatively slow growth on laboratory culture media.

### 3.5 Further *aip56* mutagenesis and detection of secondary mutations via whole‐genome sequencing

#### 3.5.1 Mutagenesis clones used for sequencing

**Figure 5:**
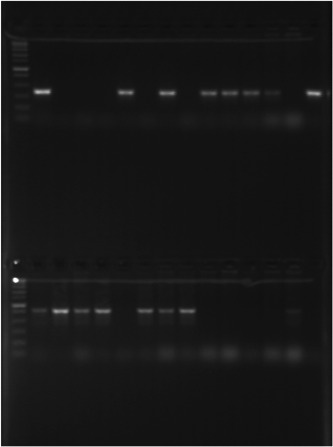
Colony PCR screening of clones selected from (kanamycin‐resistant/sucrose‐sensitive) *aip56*‐minicircle meroploid cultures on sucrose agar. Amplicons in the corresponding top and bottom parts of the gel are derived from the same clone; the top part of the gel contains *aip56*‐specific products (aip56_F/R) absent in knockout clones and the bottom part contains *kanR2*‐specific products absent in clones that reverted to WT genotype. The marker in the first lane is Fast DNA Ladder (NEB) with 10, 5, 3, 2, 1.5, 1 (reference), 0.766, 0.5, 0.3, 0.15, and 0.05 kb bands.

We aimed to confirm that electroporation of KO minicircles into sucrose‐treated competent cells can indeed generate highly isogenic KO mutants. However, sequencing of a single KO clone is not sufficient for this purpose as secondary mutations can occur during culturing either completely at random or can be associated with a particular selective pressures or/and inactivation of a particular gene (a target mutation). Thus, we obtained more *aip56* KO mutants from several independent broth cultures of a single meroploid clone (clone used to select the initial KO strain). Also, from the same individual broth cultures we have obtained clones that regained the WT genotype (“revertants”), which originate in single‐crossover cultures in the same manner as KO clones except for the second crossover happening on the same rather than the opposite homology arm. Since our meroploid contained *sacB* counterselectable marker conferring sucrose sensitivity, it was possible to select both kinds of clones in parallel on the sucrose agar, which was followed by plating on Km agar to differentiate between revertant/WT and KO genotypes (kanamycin‐sensitive and kanamycin‐resistant phenotype respectively). Plenty of clones were no longer sensitive to sucrose in three out of four nonselective meroploid broth cultures, and 30%–53% of the clones selected on sucrose were resistant to Km (Table 4). In most cases, PCR screening of selected clones for the presence of *aip56* and *kanR2* genes has confirmed either revertant to WT genotype (positive for *aip56/*negative for *kanR2*) or a KO genotype (negative for *aip56/*positive for *kanR2*) (Table 4, Figure 5). To detect and track secondary mutations, we sequenced 11 clones generated during mutagenesis: three pairs of KO and revertant clones selected on sucrose from three independent cultures, the initial *aip56* KO clone selected on sucrose/kanamycin agar, and four meroploid clones selected on Km: two from ds *aip56*‐minicircle transformation and two from ss *aip56*‐minicircle transformation (Table 5).

**Table 4:** Selection of *aip56* KO mutants and revertants to WT from independent nonselective broth cultures of a single meroploid clone QMA0786.

Number of clones	Culture 1	Culture 2	Culture 3	Cultures 4–6
Selected on sucrose plates	~50	~100	~50	None
Picked and patched on sucrose plates and kanamycin plates	14	13	15	‐
Resistant to kanamycin	5	4	8	‐
PCR‐screened for *aip56* gene and *kanR2* gene	5	4	10	‐
PCR‐positive for *aip56/*PCR‐negative for *kanR2* (revertants to WT)	2	1	4	‐
PCR‐negative for a*ip56/PCR*‐positive for *kanR2* (KOs)	2	2	3	‐

Abbreviations: KO, knockout; WT, wild type.

**Table 5:** Sequenced clones generated during *aip56* mutagenesis.

Strain	Genotype	Parent strain	Selection	Accession numbers
QMA0505	WT	‐	N/A	SRR25336394
QMA0648	KO	QMA0786	Sucrose/kanamycin	SRR25322645
QMA0780	Revertant (WT)	QMA0786	Culture 1	SRR25337471
			Sucrose	SRR25316070
				JAUYUZ000000000
QMA0781	Revertant (WT)	QMA0786	Culture 2	SRR25337470
			Sucrose	SRR25316069
				JAUYVA000000000
QMA0782	Revertant (WT)	QMA0786	Culture 3	SRR25322644
			Sucrose	
QMA0783	KO	QMA0786	Culture 1	SRR25337469
			Sucrose	SRR25316068
				JAUYVB000000000
QMA0784	KO	QMA0786	Culture 2	SRR25337468
			Sucrose	SRR25316067
				JAUYVC000000000
QMA0785	KO	QMA0786	Culture 3	SRR25337467
			Sucrose	SRR25316066
				JAUYVD000000000
QMA0786	Meroploid	QMA0505	ds‐MC transformation kanamycin	SRR25322643
QMA0787	Meroploid	QMA0505	ds‐MC transformation kanamycin	SRR25322642
QMA0788	Meroploid	QMA0505	ss‐MC transformation kanamycin	SRR25322641
QMA0789	Meroploid	QMA0505	ss‐MC transformation kanamycin	SRR25322640

Abbreviations: ds, double‐stranded; KO, knockout; N/A, not available; ss, single‐stranded; WT, wild type.

#### 3.5.2 Secondary mutations

**Figure 6:**
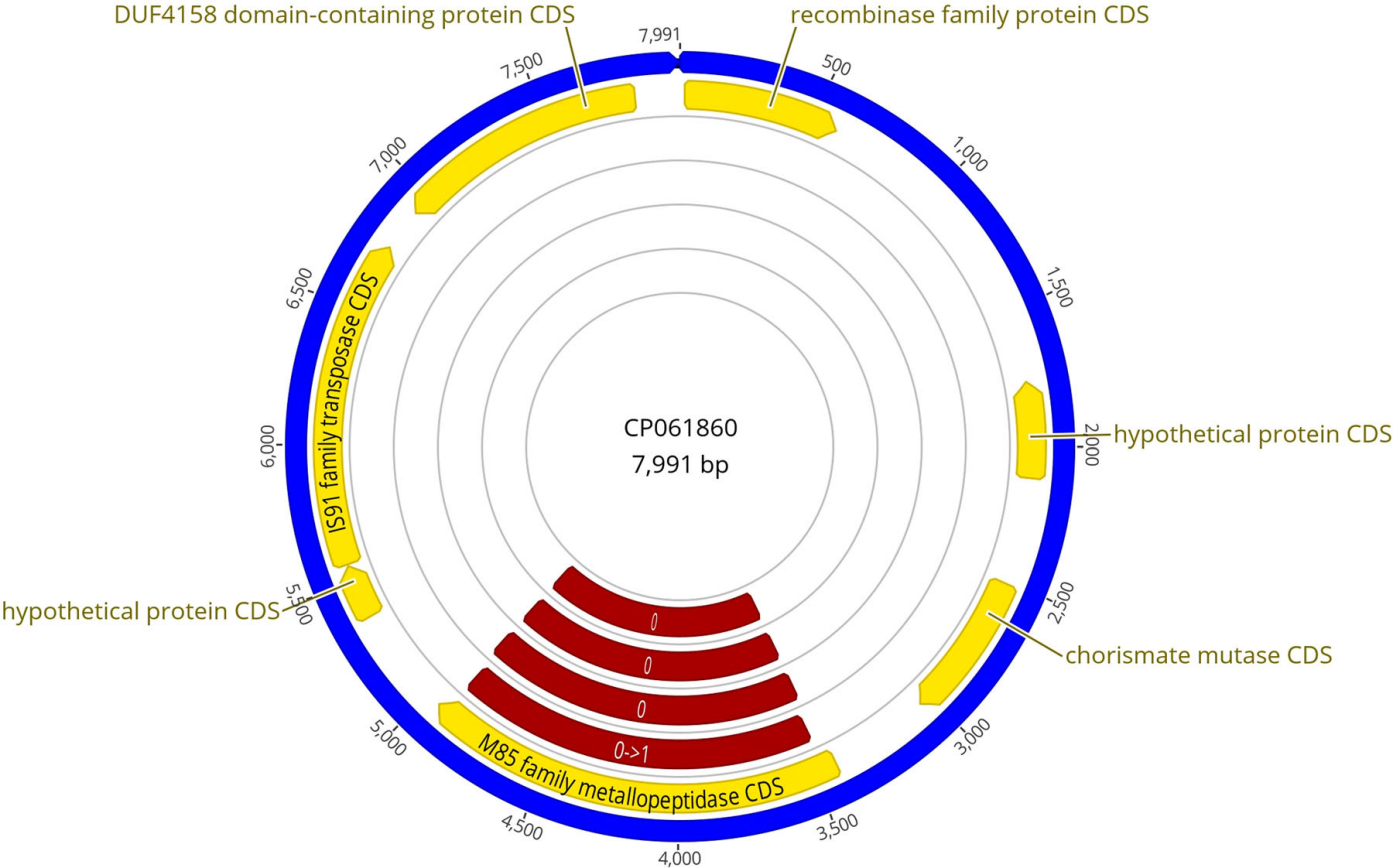
No read coverage area in reference‐guided assemblies of the knockout clones confirming *aip56* deletion. CDS, protein coding sequence.

Short reads obtained from Illumina sequencing (SRR25322640–45, SRR25336394, SRR25337467–70) were mapped onto WT reference genome CP061854–60 (Baseggio et al., 2021) to detect small variants and regions of no read coverage indicating putative large deletions. Except for two de novo substitutions in one of the ss‐minicircle transformation meroploid clones, all small mutations were variations already existing within the original cell population that increased in frequency (Tables 5 and 6). Small mutations appeared to be due to random replication errors, unlikely to have functional significance, and were mostly copy‐number variations within repetitive sequence regions. In contrast, several de novo large deletions were found in KO and revertant clones selected on sucrose plates (Tables 5 and 7). They were evident as areas of no read coverage such as in the case of intended *aip56* deletion (Figure 6), and were subsequently confirmed via long‐read Nanopore sequencing (SRR253160‐66; JAUY(VA‐D, UZ)000000000). Five out of six sucrose‐selected clones contained one large deletion, which was unique and not linked to broth culture history, that is, different variants present in KO and revertant clones selected in parallel from the same culture (Tables 5 and 7). These five deletions were flanked by transposable elements (TE), which are recognized as primary mediators of evolution in Pdp. Indeed, TE movement is promoted by stress (Twiss et al., 2005) and 15% sucrose in the medium creates high‐osmolarity environment stressful to bacteria (Cesar et al., 2020). On the other hand, the KO clone originally selected on Km/sucrose agar did not have any large deletion, which may be incidental or somehow linked to the presence of Km in the medium. Unlike the small variants, the identified large deletions ranging from 8.5 to 25.2 kb were likely to be phenotypically significant. In particular, two sucrose‐selected *aip56* KO clones, QMA0784 and QMA0785, contained deletions of the same genomic region except for a 3 kb difference. These were acquired independently as they were absent in both QMA0786 meroploid parent strain and two “sister” revertant clones QMA0781 and QMA0782 (Tables 5 and 7). Such independent deletions of the 22/25 kb sequence, which includes several known important genes happening twice, suggest that this change may be favored in *aip56*‐deficient mutants cultured on agar with high sucrose concentrations.

**Table 6:** Small secondary mutations identified in *aip56* mutagenesis clones.

Mutation	Reference nucleotides	CDS/location	Protein effect	Strains	De novo
(Tandem repeat) deletion	C1	16S rRNA	‐	QMA0787	No
(GG)3 > (GG)2	25,133–25,134				
(Tandem repeat) deletion	C1	Group II intron reverse transcriptase/maturase	Frameshift	QMA0780	No
(AA)3 > (AA)2	96,594–96,595				
Substitution	C1	IS91 family transposase	Substitution	QMA0788	Yes
A > T	149,093		D > E		
Substitution	C1	IS91 family transposase	Substitution	QMA0788	Yes
G > T	149,097		P > H		
(Tandem repeat) deletion	C1	Outside CDS	‐	QMA0648	No
(ACTCGCTT)12 > (ACTCGCTT)10	760,176–760,191			QMA0780	
				QMA0785	
				QMA0789	
(Tandem repeat) insertion	C1	Outside CDS		QMA0781	No
(TCGCCAC)9 > (TCGCCAC)10	846,596			QMA0784	
Deletion	C1	Hypothetical protein	Frameshift	QMA0781	No
−TC	858,221–858,222				
(Tandem repeat) insertion	C1	Bifunctional methylenetetrahydrofolate dehydrogenase/methenyltetrahydrofolate cyclohydrolase FolD	Truncation	QMA0784	No
(TCGCTAC)12 > (TCGCTAC)14	864,195			QMA0787	
				QMA0789	
(Tandem repeat) insertion	C1	Outside CDS	‐	QMA0782	No
(GAGGTTTC)9 > (GAGGTTTC)10	2,529,776			QMA0784	
				QMA0788–89	
Substitution/deletion	C2	Outside CDS	‐	QMA0648	No
CT > G−	744,231–744,232			QMA0782–88	

Abbreviations: CDS, protein coding sequence; rRNA, ribosomal RNA.

**Table 7:** Large deletions identified in *aip56* mutagenesis clones.

Mutation	Reference nucleotides	CDS within product/locus tag		Strains
		IC627_21610	Hypothetical protein	
Deletion 1	C2	IC627_21615	IS1‐like element ISPda1 family transposase	QMA0780
		IC627_21620	IS3 family transposase	
11, 287 bp	1,109,902–1,121,188	IC627_21625	IS3 family transposase	(revertant, culture1)
		IC627_21630	Site‐specific integrase	
		IC627_21635	Phosphoribosyltransferase	
		IC627_21640	DNA‐protecting protein DprA	
		IC627_21645	Recombinase family protein	
		IC627_21650	DUF3404 domain‐containing protein	
		IC627_17620	IS1‐like element ISPda1 family transposase	
Deletion 2	C2	IC627_17625	Type VI secretion system tip protein VgrG	QMA0781
		IC627_17630	IS91‐like element ISPda2 family transposase	
26, 434 bp	368,062–394,495	IC627_17635	Hypothetical protein	(revertant, culture2)
		IC627_17640	Hcp family type VI secretion system effector	
		IC627_17645	Penicillin‐binding protein 1B	
		IC627_17650	Exoribonuclease R	
		IC627_17655	Molybdopterin adenylyltransferase	
		IC627_17660	Isoprenylcysteine carboxylmethyltransferase family protein	
		IC627_17665	M48 family metallopeptidase	
		IC627_17670	Hypothetical protein	
		IC627_17675	IS1‐like element ISPda1 family transposase	
		IC627_17680	Fic family protein	
		IC627_17685	IS1‐like element ISPda1 family transposase	
		IC627_17690	Hypothetical protein	
		IC627_17695	DUF3010 family protein	
		IC627_17700	DEAD/DEAH box helicase	
		IC627_17705	Hypothetical protein	
		IC627_17710	Leucine‐rich repeat domain‐containing protein	
		IC627_17715	DUF3820 family protein	
		IC627_17720	NUDIX domain‐containing protein	
		IC627_17725	Galactosyl transferase	
		IC627_17730	Cation:proton antiporter	
		IC627_17735	NAD(P)H‐dependent oxidoreductase	
		IC627_17740	Iron‐containing alcohol dehydrogenase	
		IC627_17745	Gfo/Idh/MocA family oxidoreductase	
		IC627_17750	Hypothetical protein	
		IC627_17755	tRNA (pseudouridine(54)‐N(1))‐methyltransferase TrmY	
		IC627_17760	IS1‐like element ISPda1 family transposase	
		IC627_17765	DUF2955 domain‐containing protein	
		IC627_17770	HlyD family secretion protein	
		IC627_20615	NAD(P)H‐hydrate epimerase	
Deletion 3	C2	IC627_20620	Hypothetical protein	QMA0783
		IC627_20625	hypothetical protein	
8, 547 bp	927,902–936,451	IC627_20630	Hypothetical protein	(KO, culture 1)
		IC627_20635	IS1 family transposase	
		IC627_20640	IS1‐like element ISPda1 family transposase	
		IC627_20645	SCO family protein	
		IC627_20650	DUF368 domain‐containing protein	
		IC627_20655	IS1 family transposase	
		IC627_20660	IS1 family transposase	
		IC627_20665	IS1‐like element ISPda1 family transposase	
		IC627_20670	Hypothetical protein	
		IC627_20675	Glyoxalase	
		IC627_20680	IS1 family transposase	
		IC627_05605	IS1 family transposase	
Deletion 4	C1	IC627_05610	Hypothetical protein	QMA0784
		IC627_05615	DUF2947 domain‐containing protein	
25, 214 bp	1,148,799–1,174,012	IC627_05620	NAD‐dependent succinate‐semialdehyde dehydrogenase	(KO, culture 2)
		IC627_05625	NUDIX domain‐containing protein	
		IC627_05630	IS1‐like element ISPda1 family transposase	
		IC627_05635	Phosphatase PAP2 family protein	
		IC627_05640	FAD‐binding oxidoreductase	
		IC627_05645	Aquaporin Z	
		IC627_05650	LysR family transcriptional regulator	
		IC627_05655	Cation transporter	
		IC627_05660	NYN domain‐containing protein	
		IC627_05665	CG2 omega domain protein	
		IC627_05670	YggN family protein	
		IC627_05675	Hypothetical protein	
		IC627_05680	Phospholipase A	
		IC627_05685	YgiQ family radical SAM protein	
		IC627_05690	IS1‐like element ISPda1 family transposase	
		IC627_05695	YgiQ family radical SAM protein	
		IC627_05700	AAA family ATPase	
		IC627_05705	IS1‐like element ISPda1 family transposase	
		IC627_05710	IS1‐like element ISPda1 family transposase	
		IC627_05715	IS1‐like element ISPda1 family transposase	
		IC627_05720	IS1‐like element ISPda1 family transposase	
		IC627_05725	Hypothetical protein	
		IC627_05730	IS1‐like element ISPda1 family transposase	
		IC627_05735	Hypothetical protein	
		IC627_05740	Hypothetical protein	
		IC627_05745	IS1‐like element ISPda1 family transposase	
		IC627_05615	DUF2947 domain‐containing protein	
Deletion 5	C1	IC627_05620	NAD‐dependent succinate‐semialdehyde dehydrogenase	QMA0785
22, 213 bp	1,149,105–1,171,327	IC627_05625	NUDIX domain‐containing protein	(KO, culture 3)
		IC627_05630	IS1‐like element ISPda1 family transposase	
		IC627_05635	Phosphatase PAP2 family protein	
		IC627_05640	FAD‐binding oxidoreductase	
		IC627_05645	Aquaporin Z	
		IC627_05650	LysR family transcriptional regulator	
		IC627_05655	Cation transporter	
		IC627_05660	NYN domain‐containing protein	
		IC627_05665	CG2 omega domain protein	
		IC627_05670	YggN family protein	
		IC627_05675	Hypothetical protein	
		IC627_05680	phospholipase A	
		IC627_05685	YgiQ family radical SAM protein	
		IC627_05690	IS1‐like element ISPda1 family transposase	
		IC627_05695	YgiQ family radical SAM protein	
		IC627_05700	AAA family ATPase	
		IC627_05705	IS1‐like element ISPda1 family transposase	
		IC627_05710	IS1‐like element ISPda1 family transposase	
		IC627_05715	IS1‐like element ISPda1 family transposase	
		IC627_05720	IS1‐like element ISPda1 family transposase	

Abbreviations: CDS, protein coding sequence; KO, knockout.

Thus, it appears that prolonged exposure to high sucrose concentrations used for *sacB* counterselection might have created stressful conditions, which activated TE movement in *Pdp*. This indicates that alternative negative selection markers should be considered for TE‐rich bacteria, and may potentially allow to obtain a viable ssrA‐smpB mutant in *Pdp*. In contrast, preparation of the competent cells using high sucrose buffer and electroporation and integration of the allelic exchange DNA in the form of the minicircles had virtually no mutagenic effect (even when minicircles were delivered as ssDNA).

## 4 CONCLUSIONS

We describe donor‐ and vector‐free allelic exchange knockout employing “minicircles” in nonstandard epidemiologically relevant bacterial pathogen *Photobacterium damselae subsp. piscicida* (*Pdp*). *Pdp* is not readily transformable by standard techniques that involve competent cell preparations using hypertonic saline buffers (Cutrín et al., 1995). As a result, delivery of the mutagenic DNA via conjugation of pir/RP4 suicide vectors from SM10/S17‐1 λ pir donor strains of *E. coli* has been used in previous genetic modifications of *Pdp*, a method notorious for the high rate of the off‐target mutation (Babic et al., 2008; Ferrières et al., 2010; Strand et al., 2014). Here we have achieved high transformation efficiency comparable to commercial *E. coli* preparations via a simple single‐buffer single‐ingredient protocol. This method avoids salt and uses concentrated sucrose solution as an all‐in‐one hypertonic wash buffer, cryoprotecting medium, and electroporation buffer. Nonionic osmotic pressure stabilizes membranes for electroporation and, unlike ions from salts, does not interfere with electric discharge and does not contribute to DNase activity (Wang & Griffiths, 2009). Potentially, this approach may be generally efficient in bacteria as it was successfully used in other Vibrionaceae (Wang & Griffiths, 2009) and phylogenetically distant Streptococcaceae (Framson et al., 1997).

**Figure 7:**
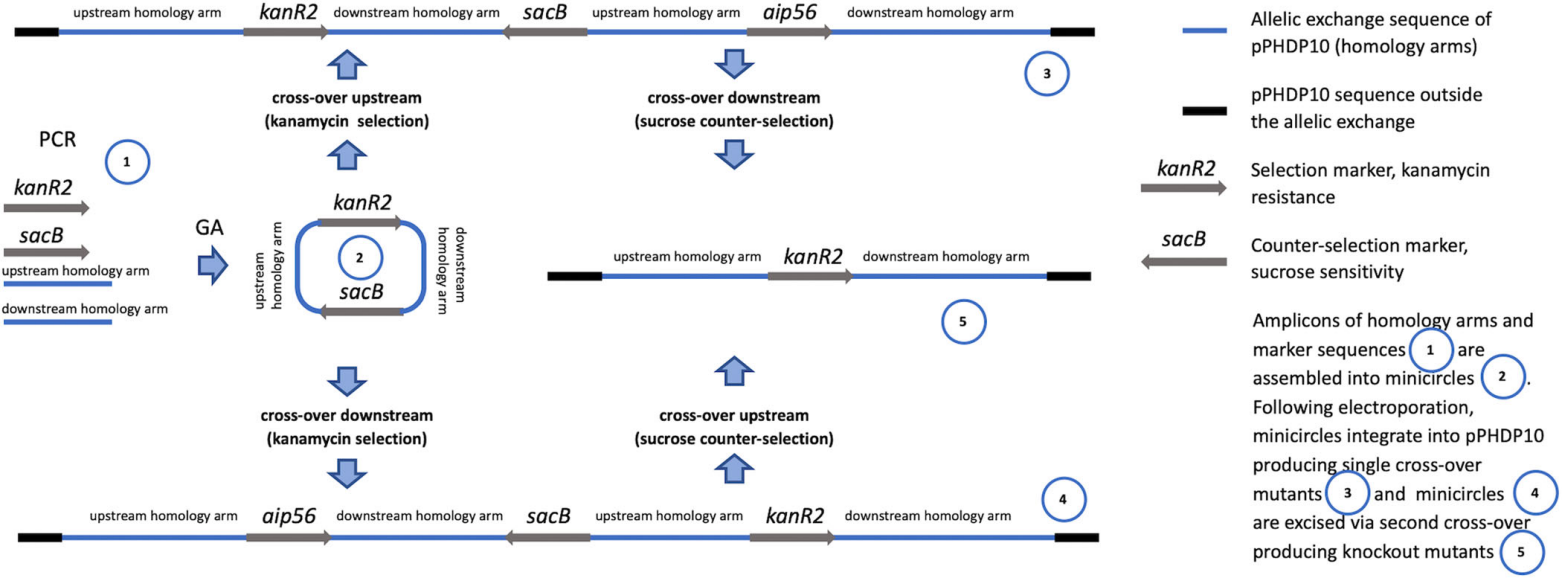
Schematic representation of *aip56* knockout. GA, Gibson assembly.

High‐efficiency transformation is required for allelic exchange mutagenesis using nonreplicating DNA. The latter is conventionally an allelic exchange construct (AEC) cloned in a suicide vector. However, plasmid backbones increase the chances of secondary mutations (Desomer et al., 1991; Li et al., 2018; Oliveira et al., 2010; de Vries & Wackernagel, 2002). Here we have mutated *Pdp* via vector‐free allelic exchange employing KO minicircles, nonreplicating circular minimalistic AEC containing only mutagenesis essentials that were generated by GA (Gibson et al., 2009). Electroporated KO minicircles targeting *aip56* and *ssrA‐smpB* loci readily integrated into plasmid and chromosomal DNA, respectively, evident by highly efficient selection of single‐crossover mutants. Furthermore, KO mutants of the *aip56* gene encoded on highly abundant small (<10 kb) plasmid were selected with ease. This was potentially facilitated by the minicircle approach, as integration of a longer sequence containing a suicide vector backbone is likely to affect host plasmid stability and/or replication. We summarize the employed method using *aip56* KO as an example below, which is schematically represented in Figure 7: *aip56‐*KO minicircle of 4.7 kb (0.9 kb sequence upstream from *aip56* ‐> selectable marker gene (*kanR2*, Km resistance, 0.8 kb) ‐> 0.9 kb sequence downstream from *aip56* ‐> counterselectable marker transcript (*sacB*, sucrose sensitivity, 2.1 kb) was generated by GA (Figure 1) and electroporated into *Pdp* cells rendered competent using highly concentrated sucrose solution (as described in subsection 3.1). Single‐crossover mutants were selected by the gain of Km resistance, and confirmed by PCR from Km gene to genomic sequence outside the homology arms (Figure 3a,b), along with the gain of sucrose sensitivity. Double‐crossover mutants were selected by loss of sucrose sensitivity, then confirmed by the lack of *aip56*‐specific PCR amplification and by PCR across allelic exchange locus yielding shorter product than in the WT parent, accounting for the size difference of *aip56* and *kanR2* (Figure 4).

Whole‐genome sequencing of multiple clones generated during *aip56* mutagenesis did not reveal any secondary mutations that could be attributed to sucrose treatment used to render the cells competent or electroporation/integration of minicircles (Tables 5, 6, 7). However, it identified several large deletions associated with the mobilization of TEs by prolonged exposure to high sucrose concentrations used for *sacB* counterselection (Tables 6 and 7). In this respect, our vector‐free method is highly flexible as minicircles can be designed/assembled to contain alternative selection markers or/and to generate other kinds of mutations. To conclude, electroporation of minicircles into sucrose‐treated cells is an efficient and versatile approach for allelic exchange allowing to generate highly isogenic mutants in *Pdp* and potentially most of the bacterial species.

## AUTHOR CONTRIBUTIONS

**Oleksandra Rudenko**: Conceptualization (lead); investigation (lead); methodology (lead); validation (lead); visualization (lead); writing—original draft (lead); writing—review and editing (equal). **Laura Baseggio**: Conceptualization (supporting); investigation (supporting); methodology (supporting); validation (supporting); writing—review and editing (equal). **Fynn McGuigan**: Investigation (supporting); methodology (supporting); validation (supporting); writing—review and editing (equal). **Andrew C. Barnes**: Conceptualization (supporting); funding acquisition (lead); resources (lead); writing—review and editing (equal).

## CONFLICT OF INTEREST STATEMENT

None declared.

## ETHICS STATEMENT

None required.

## Data Availability

Sequencing data were made available under BioProject PRJNA994685, https://www.ncbi.nlm.nih.gov/bioproject/PRJNA994685, which includes deposition of Illumina reads (SRR25322640‐45, SRR25336394, SRR25337467‐70), and MinION reads (SRR253160‐66) and assemblies (JAUYUZ000000000, JAUYVA000000000, JAUYVB000000000, JAUYVC000000000, JAUYVC000000000).
